# Cyst infection in autosomal dominant polycystic kidney disease: our experience at Toranomon Hospital and future issues

**DOI:** 10.1007/s10157-020-01928-2

**Published:** 2020-07-22

**Authors:** Tatsuya Suwabe

**Affiliations:** 1 Department of Nephrology, Toranomon Hospital Kajigaya, 1-3-1 Kajigaya, Takatsu-ku, Kawasaki-shi, Kanagawa-ken, 213-0015 Japan; 2 Okinaka Memorial Institute for Medical Research, Toranomon Hospital, 2-2-2 Toranomon, Minato-ku, Tokyo, Japan

**Keywords:** ADPKD, Cyst infection, Infected cyst, Polycystic kidney disease

## Abstract

Cyst infection is a frequent and serious complication of autosomal dominant polycystic kidney disease (ADPKD) that is often difficult to treat and can be fatal. However, much is still unknown about cyst infection. Positron emission tomography (PET) is generally recommended for detecting infected cysts, but it has the disadvantages of limited availability, high cost, and radiation exposure. We have devised magnetic resonance imaging (MRI) diagnostic criteria for cyst infection. Lipid-soluble antibiotics such as fluoroquinolones show good penetration into cysts and are recommended for cyst infection. However, we reported that fluoroquinolone-resistant microorganisms showed a high prevalence in cyst infection. We should, therefore, reconsider the empirical use of fluoroquinolones for ADPKD patients with cyst infection. We have suggested a new antibiotic strategy according to the severity of cyst infection. It may be important to consider the drug half-life in serum in addition to the drug susceptibility when selecting antibiotics Cyst drainage is necessary for some patients with refractory cyst infection; however, cyst drainage can be associated with severe adverse events. We suggest adaptation criteria for cyst drainage in patients with cyst infection in ADPKD. Most causative bacteria of cyst infection are enterobacteria, and hematogenous spread via bacterial translocation in the intestine is considered the main cause of cyst infection. Therefore, intestinal flora may be important for cyst infection. The role of the intestinal flora in cyst infection in ADPKD is unknown and should be explored in future research.

## Introduction

Autosomal dominant polycystic kidney disease (ADPKD) is a common inherited renal disorder [1, 2], and cyst infection is a frequent and serious complication of ADPKD. It has been estimated that 30–50% of patients with ADPKD experience some form of renal infection during their lifetime [3, 4], although cyst infection leading to hospitalization is much less frequent, occurring in approximately 9% of cases [5]. These infections sometimes become resistant and can be fatal, even when appropriate antibiotics are administered [5–7]. However, much is still unknown about cyst infection in ADPKD, and there are no evidence-based guidelines for the management of cyst infection in these patients [8]. The limited evidence currently available also makes it difficult to identify risk factors for treatment failure [9].

In this article, recent evidence concerning the diagnosis and therapeutic options of cyst infection in cases of ADPKD is reviewed. The clinical features of cyst infection in ADPKD of Japan are also described, and we consider the etiology and origin of cyst infection. We further suggest diagnostic and therapeutic strategies of cyst infection according to our experience in Toranomon Hospital and discuss issues associated with this disease that must be managed in the future.

### Clinical features of patients with ADPKD in Japan

In Japan, about 10,000 ADPKD patients are on dialysis, and very few receive kidney or liver transplantation because of a severe donor shortage. This might have increased the proportion of long-term dialysis patients with massive renomegaly or hepatomegaly in our study population. For massive enlarged kidney or liver, we perform renal or hepatic transcatheter arterial embolization (TAE). The total kidney volume decreases by about 45% on average at 1 year after renal TAE [10], while the liver volume decreases by about 9.2% on average [11]. The effectiveness of hepatic TAE is limited, which is why markedly fewer patients receive hepatic TAE than renal TAE. In contrast, renal TAE is currently performed worldwide, and various embolic agents are reported to be effective [12–16]. Renal TAE is also conducted before renal transplantation to obtain a sufficient volume reduction for graft implantation [17]. Renal TAE has thus become a standard option for the treatment of enlarged kidneys in ADPKD in Japan.

Cyst infection is a relatively frequent complication in ADPKD patients, especially in patients on long-term dialysis with hepatomegaly in Japan. For example, a total of 492 ADPKD patients (2278 episodes) were admitted to Toranomon Hospital with a diagnosis of cyst infection during the period from January 2002 to December 2019. The number of patients with ADPKD who were referred to our hospital was 2602 persons (4461 episodes) aside from cases of scheduled hospitalization for evaluations after renal TAE, so the number of patients hospitalized by cyst infection accounted for 18.9% of all ADPKD patients referred to our hospital, and the number of cases of hospitalization due to cyst infection accounted for 51.1% of all hospitalized episodes in patients with ADPKD in our hospital. Some patients were admitted several times due to cyst infection. One patient was admitted more than 50 times with a diagnosis of cyst infection since 2004 in our hospital. Renal cyst infection may unlikely occur after renal TAE [5, 19], hepatic cyst infection, by contrast, is a much more serious problem in Japan.

### Imaging modalities for the diagnosis of cyst infection in Japan

Positron emission tomography-computed tomography (PET-CT) is generally recommended for detecting infected cysts [5], but this imaging method has disadvantages of limited availability and high cost. In addition, it was reported that the radiation dose associated with PET might be increased in patients with renal failure [18]. In addition, it is not covered by health insurance for the diagnosis of cyst infection in Japan, and patients must therefore pay the high costs of PET-CT out of their own pocket, making it difficult to perform PET in ADPKD patients whenever cyst infection is suspected. By contrast, MRI is covered by health insurance and can be very convenient for patients on dialysis in terms of the cost, as their medical expenses are much more thoroughly supported by the government than non-dialysis patients. In addition, the number of MRI scanners per million population in Japan was about 50 as of 2017, which was the highest number in the world at that time [19]. In contrast, the number of PET per million population in Japan is only about 5. MRI is therefore much more convenient than PET-CT in terms of cost and availability in Japan. For this reason, we usually use MRI to diagnose infected cysts. However, there are some issues associated with using MRI for the identification of infected cysts. First, acute or chronic intracystic hemorrhaging is often seen in polycystic kidneys. It may be more difficult to differentiate infected renal cysts and these sites of acute or chronic intracystic hemorrhaging by MRI than PET-CT [20], which may be why the features of infected cysts on MRI are generally less specific than those on PET-CT. However, intracystic hemorrhaging in renal cysts is very unlikely to occur in patients who have received renal TAE, and given that the majority of our patients have received renal TAE, renal intracystic hemorrhaging is rare in this population. Therefore, the features of infected cysts on MRI may be more specific in Japan than in other countries.

### Identification of infected cysts in ADPKD

**Fig. 1:**
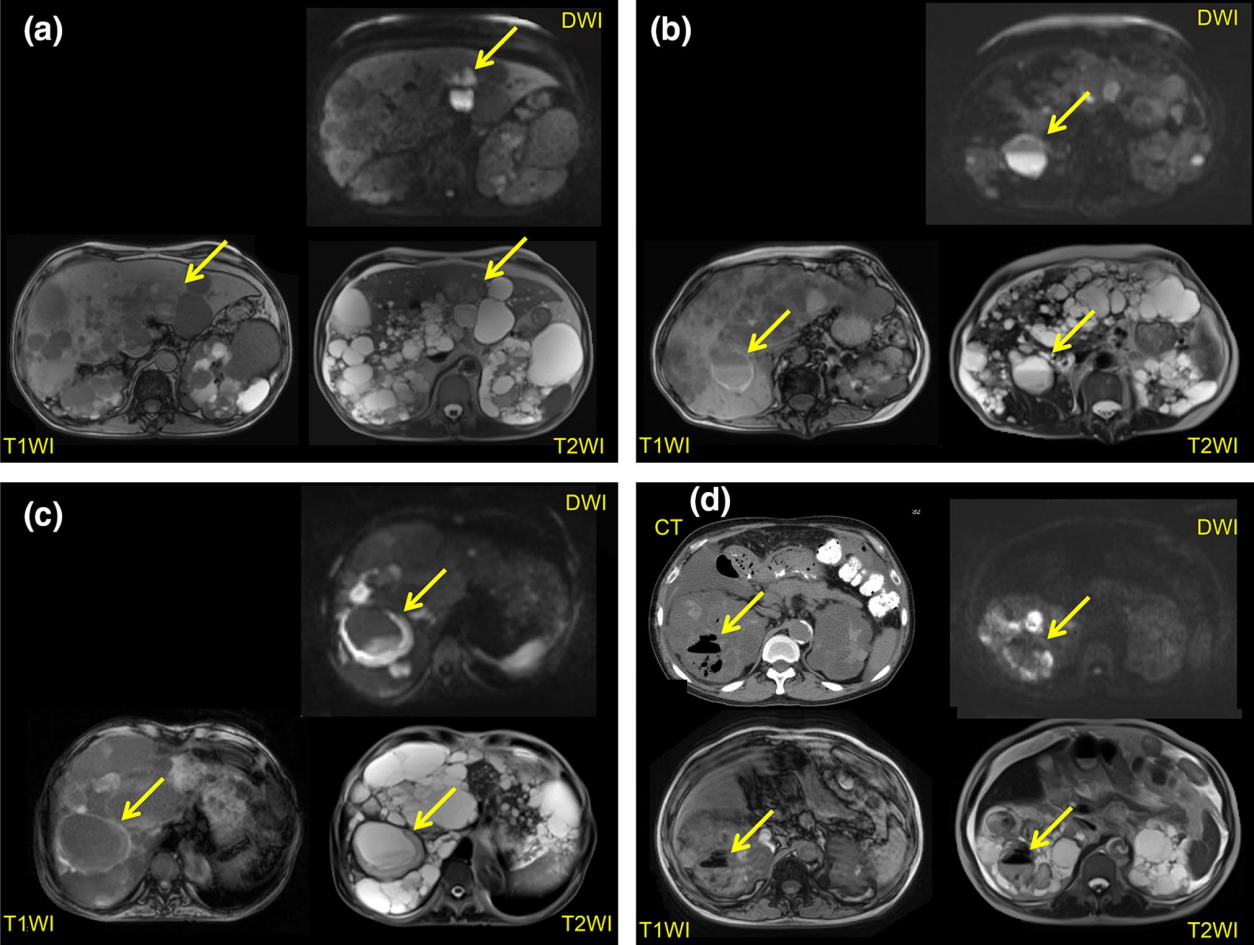
**a** MRI findings (T1WI, T2WI, and DWI) in a patient with cyst infection. The infected renal cyst shows a higher intensity on DWI compared with normal cysts, but it is difficult to identify on T1WI and T2WI. **b** MRI findings (T1WI, T2WI, and DWI) in a patient with cyst infection. A fluid-fluid level and cyst wall thickening can be seen. The infected renal cyst shows a higher intensity on DWI and T1WI than normal cysts, while it has a lower intensity on T2WI. **c** MRI findings (T1WI, T2WI, and DWI) in a patient with cyst infection. Obvious cyst wall thickening can be seen. The infected cyst is iso-intense on T1WI, T2WI, and DWI. **d** MRI findings (T1WI, T2WI, and DWI) in a patient with cyst infection. Gas is seen on T1WI, T2WI, and CT. The infected renal cyst shows a higher intensity on DWI compared with normal cysts, while it has a lower intensity on T2WI and T1WI. Sited from Suwabe T, et al. BMC Nephrol. 2016 Nov 9;17(1):170

We recently devised MRI diagnostic criteria for cyst infection (Additional file 1) [20]. The possibility of cyst infection can be assessed using our criteria even if causative microorganisms are not identified by culture of the cyst contents [21]. In our criteria, the detection of four intracystic features on abdominal MRI (a high signal intensity [SI] on diffusion-weighted imaging [DWI], fluid-fluid level, wall thickening, and gas) and the assessment of changes over time are important for detecting cyst infection (Fig. 1a–d). The disadvantage of using MRI to detect infected cysts is the low specificity, especially in patients with organomegaly [22]. Intracystic gas is specific for cyst infection, but its sensitivity is only 1.1% (Table 1). A high intracystic SI on DWI showed a sensitivity of 86.4%, but its specificity was relatively low at 33.3% (Tables 2, 3). Both the specificity and sensitivity of a fluid–fluid level and wall thickening were about 80%. However, the specificity of these MRI features decreased as the total liver and kidney volume (TLKV) increased, falling to 65.8% in patients with organomegaly (TLKV > 8500 cm^3^). A cyst diameter > 5 cm was deemed useful for detecting severely infected cysts that needed drainage, and the specificity was increased by combining it with the other four MRI findings. We concluded that we could identify most cases of severe cyst infection that required drainage by combining the 4 MRI features with abdominal pain at the same location plus changes from previous MRI findings and a cyst diameter >5 cm with almost 100% sensitivity and at least 84.4% specificity.

**Table 1:** Number of episodes with each MRI feature of cyst infection, and the sensitivity and specificity of each MRI feature

	Cases (*n* = 88)	Controls (*n* = 147)	Sensitivity	Specificity
High SI on DWI (%)	86.4	66.7	86.4	33.3
Fluid-fluid level (%)	50.0	12.9	50.0	87.1
Wall thickening (%)	48.3	10.9	48.3	89.1
Fluid-fluid level or wall thickening (%)	84.1	19.7	84.1	80.3
Gas (%)	1.1	0	1.1	100
At least one of these four features (%)	100	68.0	100	32.0
High SI on DWI with diameter > 5 cm (%)	69.3	15.6	69.3	84.4
Fluid-fluid level or wall thickening with diameter > 5 cm (%)	72.7	8.8	72.7	91.2
At least one of these four features with diameter > 5 cm (%)	83.0	18.4	83.0	81.6

Sited from Suwabe et al. BMC Nephrol (2016) 9;17(1):170

**Table 2:** Number of episodes in cases with positive MRI features of cyst infection and intracystic changes, and the sensitivity of each MRI feature and intracystic changes in four groups stratified by TKLV

	All episodes in cases (*n* = 88)	Cases with TKLV < 3000 cm^3^ (*n* =13)	Cases with TKLV 3000 to < 5500 cm^3^ (*n* = 39)	Cases with TKLV 5500 to 8500 cm^3^ (*n* = 17)	Cases with TKLV >8500 cm^3^ (*n* =17)	*p* value
Number of episodes M/F)	88 (39/49)	13 (0/13)	39 (16/23)	17 (8/9)	17 (14/3)	<0.0001
Age	64.6 ± 10.7	63.4 ± 17.1	65.5 ± 11.0	66.6 ± 7.7	61.8 ± 5.8	NS
Renal function (Dialysis %)	80.2	92.3	76.9	70.6	88.2	NS
High SI on DWI (%)	86.4	92.3	82.5	82.4	94.4	NS
Fluid-fluid level (%)	50.0	38.5	55.0	58.8	38.9	NS
Wall thickening (%)	48.3	53.9	45.0	47.1	52.9	NS
Fluid-fluid level or wall thickening (%)	84.1	84.6	85.0	82.4	83.3	NS
Gas (%)	1.1	0	2.5	0	0	NS
At least one of these four features (%)	100	100	100	100	100	NS
High SI on DWI with diameter > 5 cm (%)	69.3	53.9	67.5	82.4	72.2	NS
Fluid-fluid level or wall thickening with diameter > 5 cm (%)	72.7	53.9	75.0	82.4	72.2	NS
At least one of these four features with diameter > 5 cm (%)	83.0	61.5	85.0	100.0	77.8	NS

*TKLV* total kidney and liver volume, *NS* not significant

Sited from Suwabe T, et al. BMC Nephrol*.* 2016 Nov 9;17(1):170

**Table 3:** Number of controls without MRI features of cyst infection and intracystic changes, and the specificity of each MRI feature and intracystic changes in four groups stratified by TKLV

	All controls (*n* = 147)	Controls with TKLV < 3000 cm^3^ (*n* = 39)	Controls with TKLV 3000 to < 5500 cm^3^ (*n* = 35)	Controls with TKLV 5500 to <8500 cm^3^ (*n* = 35)	Controls with TKLV >8500 cm^3^ (*n* = 38)	*p* alue
Number of patients M/F)	147 (62/85)	39 (14/25)	35 (14/21)	35 (19/16)	38 (15/23)	NS
Age (years)	53.3 ± 11.0	50.3 ± 14.7	52.3 ± 8.8	55.4 ± 9.5	55.4 ± 9.1	NS
Renal function (Dialysis %)	49.0	5.1	31.4	74.3	86.8	< 0.0001
Without high SI on DWI (%)	33.3	69.2	37.1	14.3	10.5	< 0.0001
Without fluid-fluid level (%)	87.1	100.0	82.9	82.9	81.6	< 0.01
Without wall thickening (%)	89.1	100.0	94.3	82.9	79.0	< 0.005
Without fluid-fluid level or wall thickening (%)	80.3	100.0	82.9	71.4	65.8	< 0.0001
Without gas (%)	100	100	100	100%	100	NS
None of the four features (%)	32.0	69.2	34.3	14.3	7.9	< 0.0001
Without high SI on DWI (diameter > 5 cm) (%)	84.4	100.0	94.3	77.1	65.8	0.0001
Without fluid-fluid level or wall thickening (diameter > 5 cm) (%)	91.2	100.0	94.3	94.3	76.3	< 0.005
Without at least one of the four features (diameter > 5 cm) (%)	81.6	100.0	91.4	74.3	60.5	0.0001

*TKLV* total kidney and liver volume, *NS* not significant

Sited from Suwabe T, et al. BMC Nephrol. 2016 Nov 9;17(1):170

As mentioned above, acute or chronic intracystic hemorrhaging may be the most difficult disease to differentiate from infected cysts, which decreases the specificity of MRI for the diagnosis of infected cysts. However, renal intracystic hemorrhaging rarely occurs in patients who have received renal TAE, and hepatic intracystic hemorrhaging is even rarer than renal intracystic hemorrhaging. Therefore, the specificity of features of infected cyst on MRI increases in patients who have received renal TAE. Most of the patients in our hospital have received renal TAE, and we do not usually encounter substantial problems associated with the diagnosis of cyst infection by MRI.

### Poor prognostic factors influencing cyst infection in ADPKD

We investigated the clinical factors related to the duration of hospitalization and death due to cyst infection [23]. All ADPKD patients who underwent cyst drainage, nephrectomy or partial hepatectomy at Toranomon Hospital from January 2004 to March 2016 in whom a cyst fluid analysis showed bacteria or neutrophils indicating definite cyst infection were retrospectively reviewed. A total of 243 patients with cyst infection were enrolled, including 104 men and 139 women (mean age: 62.6 ± 10.0 years). Seventeen patients died of a definite cyst infection.

A multivariate logistic regression analysis revealed that hepatomegaly, positive cyst contents in culture, and a high white blood cell (WBC) count were significantly associated with death from cyst infection. Death was significantly more likely if patients had massive hepatomegaly (≥ 5113.2 ml) compared with mild hepatomegaly (< 1898.7 ml) (odds ratio: 38.1; 95% confidence interval [CI] 2.9–492.6, *p *= 0.0004). A multivariate Cox proportional hazards analysis revealed that old age, long dialysis duration, hepatic TAE, high serum total cholesterol level, high WBC count, and hepatomegaly were significantly associated with longer hospitalization for cyst infection. Hepatomegaly was the strongest factor, being associated with both death and longer hospitalization due to cyst infection. Dialysis was not a statistically significant factor associated with death from cyst infection in this series, probably due to a lack of a sufficient number of patients. But it is generally said that patients with ESRD have disorders of the immune systems [24] and a majority of the patients with serious cyst were on dialysis. Therefore, ESRD may be a poor prognostic factor for cyst infection.

### Causative microorganisms for cyst infection and susceptibility to lipid-soluble antibiotics

**Fig. 2:**
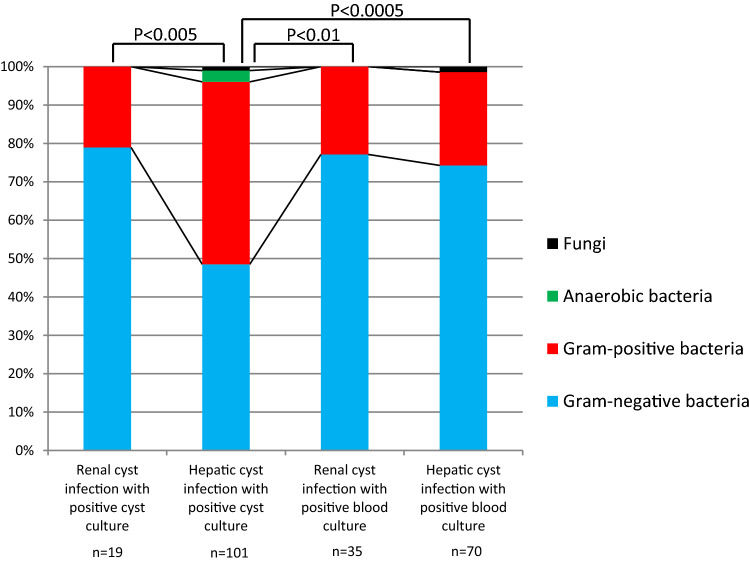
Causes of cyst infection in each group. Sited from Suwabe T, et al. Eur J Clin Microbiol Infect Dis. 2015;34(7):1369-79

We conducted a study to determine the profile of the microorganisms causing cyst infection in ADPKD and their susceptibility to lipid-soluble antibiotics [21]. This retrospective study reviewed all ADPKD patients admitted to Toranomon Hospital with a diagnosis of cyst infection from January 2004 to March 2014. All patients who underwent cyst drainage and had positive cyst fluid cultures were enrolled. Patients with positive blood cultures who satisfied our criteria for cyst infection or probable infection were also enrolled. There were 99 cases with positive cyst fluid cultures and 93 cases with positive blood cultures. Gram-negative bacteria accounted for 74–79% of the isolates in all groups, except for patients with positive hepatic cyst fluid cultures (Fig. 2). The susceptibility of *Escherichia coli* to fluoroquinolones was very low in patients with hepatic cyst infection, especially those with frequent episodes and those with hepatomegaly (Table 4). Fungi were detected in two cases. As such, fluoroquinolone-resistant microorganisms showed a high prevalence in cyst infection. This result suggests that lipid-soluble antibiotics are not always effective for cyst infection and it is important to identify causative microorganisms in order to avoid the overuse of fluoroquinolones.

**Table 4:** Susceptibility of major gram-negative causative bacteria to lipid-soluble antibiotics

	Isolates	LVFX	CPFX	TS
Cyst fluid culture in renal cyst infection	*Escherichia coli* (11 episodes in 11 patients) *	54.5% (6/11)	54.5% (6/11)	63.6% (7/11)
	*Klebsiella* (2 episodes in 2 patients)	100% (2/2)	100% (2/2)	100% (2/2)
	*Enterobacter* (*n* = 0)	NA	NA	NA
	*Pseudomonas aeruginosa* (*n *= 0)	NA	NA	NA
Cyst fluid culture in hepatic cyst infection	*Escherichia coli* (23 episodes in 20 patients) †	21.7% (5/23)	21.7% (5/23)	65.2% (15/23)
	*Klebsiella* spp. (9 episodes in 9 patients) ‡	88.9% (8/9)	88.9% (8/9)	88.9% (8/9)
	*Enterobacter* spp. (4 episodes in 4 patients)	75.0% (3/4)	75.0% (3/4)	100% (4/4)
	*Pseudomonas aeruginosa* (5 episodes in 3 patients)	60.0% (3/5)	60.0% (3/5)	NA
Blood culture in renal cyst infection	*Escherichia coli* (11 episodes in 10 patients) §	63.6% (7/11)	66.7% (6/9)	63.6% (7/11)
	*Klebsiella* (8 episodes in 8 patients)	100% (8/8)	100% (8/8)	100% (8/8)
	*Enterobacter* (2 episodes in 2 patients)	100% (2/2)	100% (2/2)	100% (2/2)
	*Pseudomonas aeruginosa* (n=0)	NA	NA	NA
Blood culture in hepatic cyst infection	*Escherichia coli* (*n* = 20) ∥	30.0% (6/20)	31.6% (6/19)	65.0% (13/20)
	*Klebsiella* spp. (13 episodes in 13 patients)	92.3% (12/13)	92.3% (12/13)	100% (13/13)
	*Enterobacter spp.* (8 episodes in 7 patietns)	87.5% (7/8)	87.5% (7/8)	100% (7/7)
	*Pseudomonas aeruginosa* (5 episodes in 5 patients)	60.0% (3/5)	60.0% (3/5)	NA

*LVFX* levofloxacin, *CPFX* ciprofloxacin, *TS* trimethoprim-sulfamethoxazole, *NA* not applicable

*Extended spectrum beta lactamase (ESBL) was detected in *E. coli* from 3 renal cyst fluid cultures

^†^ESBL was detected in *E. coli* from 6 hepatic cyst fluid cultures

^‡^ESBL was detected in *Klebsiella* from 1 hepatic cyst fluid culture

^§^ESBL was detected in *E. coli* from 4 blood culture of renal cyst infection

^∥^ESBL was detected in *E. coli* from 4 blood culture of hepatic cyst infection

Sited from Suwabe et al. Eur J Clin Microbiol Infect Dis. 2015;34(7):1369-79

### Origin of renal or hepatic cyst infection

We previously reported that most microorganisms causing cyst infection were enterobacteria [21], which is consistent with the findings of other studies [5, 25]. It is generally believed that the route of cyst infection is hematogenous spread or retrograde infection. However, fewer patients had positive urine cultures in our study than in other reports, and renal cyst infection was equally common in men and women [6, 21]. This may be because the majority of our dialysis patients who have undergone renal TAE were completely anuric, and urinary tract infection may be rare in completely anuric patients. In addition, the remarkable elevation of biliary enzymes was not seen in most episodes of hepatic cyst infection. These findings suggest that the majority of cyst infections in our study population might have been caused by hematogenous spread rather than retrograde infection. This hypothesis is supported by our case report in which *Salmonella* grew from samples of blood and cyst contents in ADPKD patients with *Salmonella* enteritidis cultured from stool samples [26].

In about half (14 of 26 episodes) of hepatic cyst infection cases, microorganisms cultured from the blood did not completely match the isolates from the cyst fluid [21]. We usually perform cyst drainage and cyst fluid culture after the administration of antibiotics for several weeks. However, blood cultures are usually performed just after the onset of cyst infection, which might explain why the cyst fluid isolates differed from those detected by blood cultures. Microorganisms detected by blood culture might reflect the ones causing early infection, while those detected by cyst fluid culture might be responsible for late infection. Invasion of intestinal microorganisms can occur repeatedly, with invasion of resistant microorganisms surviving in the intestines after antibiotic therapy leading to late infection. Accordingly, enterobacteria may play important roles in the onset of cyst infection. Further investigations on this topic are needed.

### Antibiotic therapy for cyst infection

Drug delivery is independent of glomerular filtration when cysts do not communicate with the renal tubules, so water-soluble antibiotics do not penetrate such cysts well [3, 27–29]. In contrast, lipid-soluble antibiotics show good penetration into cysts and are recommended for the treatment of infected cysts in ADPKD [3, 27, 29–33]. As mentioned above, Gram-negative rods, such as *E. coli*, are the main causative agents of cyst infection in ADPKD [5, 6, 25]. Among lipid-soluble antibiotics, fluoroquinolones and trimethoprim–sulfamethoxazole have a broad range of activity against Gram-negative rods and are generally used to treat cyst infection. Trimethoprim–sulfamethoxazole is not recommended for patients with end-stage renal disease [34], so we often select fluoroquinolones as first-line antibiotics for cyst infection. However, we reported that fluoroquinolone-resistant microorganisms showed a high prevalence in cyst infection [21].

**Fig. 3:**
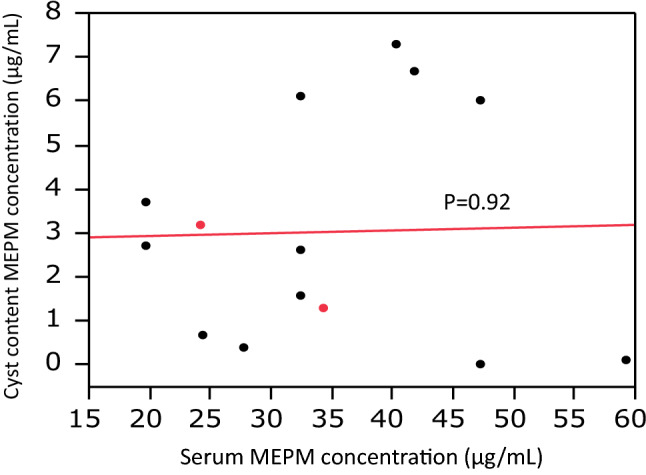
Relationship between the intracystic MEPM concentration and serum MEPM concentration. Black circles: patients with hepatic cyst infection, Red circles: patients with renal cyst infection. Sited from Hamanoue S, et al. BMC Nephrol. 2018;19(1):272

**Fig. 4:**
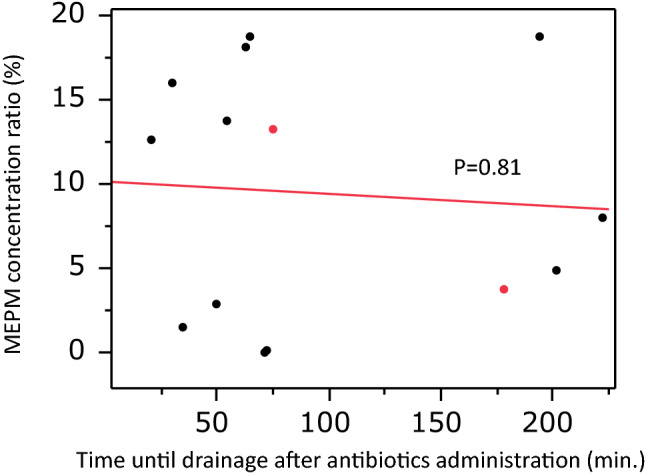
Relationship between the intracystic MEPM concentration and time to drainage after administration of MEPM. Black circles: patients with hepatic cyst infection, Red circles: patients with renal cyst infection. Sited from Hamanoue S, et al. BMC Nephrol. 2018;19(1):272

**Fig. 5:**
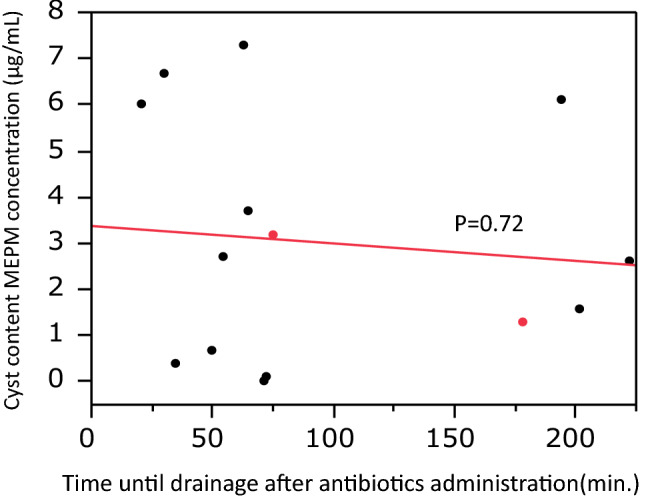
Relationship between the MEPM concentration ratio and time to drainage after administration of MEPM. Black circles: patients with hepatic cyst infection, Red circles: patients with renal cyst infection. Sited from Hamanoue S, et al. BMC Nephrol. 2018;19(1):272

Carbapenem therapy may be required for some cyst infections that are resistant to lipid-soluble antibiotics, especially in patients with ESBL-positive bacteria. Meropenem (MEPM) is a representative carbapenem with a broad spectrum of activity, but little is known about the penetration of such newer carbapenems into the cysts of ADPKD patients. Therefore, we investigated the penetration of MEPM into infected cysts of ADPKD patients [35]. Between August 2013 and January 2014, 10 ADPKD patients (14 infected cysts) receiving MEPM at Toranomon Hospital underwent drainage of infected cysts, and definite cyst infection was confirmed through the detection of neutrophils by a cyst fluid analysis. The serum concentration of MEPM was measured just after intravenous administration and compared with that in fluid aspirated from infected cysts. In the patients undergoing cyst drainage, the mean serum MEPM concentration was 35.2 ± 12.2 μg/ml (range: 19.7–59.2 μg/ml), while the mean cyst fluid concentration of MEPM in the drained liver cysts (*n* = 12) or kidney cysts (*n* = 2) was 3.03 ± 2.6 μg/ml (range: 0–7.3 μg/ml) (Fig. 3). In addition, the mean cyst fluid/serum MEPM concentration ratio was 9.46% ± 7.19% (range: 0–18.8%) (Fig. 4). There was no relationship between the cyst fluid concentration of MEPM and the time until drainage after MEPM administration or between the cyst fluid/serum MEPM concentration ratio and the time until drainage (Fig. 5). These findings suggest that MEPM shows poor penetration into infected cysts in ADPKD patients. However, MEPM is clinically effective for cyst infection in most ADPKD patients. One of the reasons for this apparent discrepancy might be that the minimum inhibitory concentration (MIC) of MEPM is relatively low for most bacteria. For example, the MIC_90_ of MEPM for *E. coli* and *Klebsiella pneumonia* (typical gram negative bacteria) was only 0.03 μg/ml according to a survey performed in Japan [36]. However, the MIC_90_ of MEPM is high for some bacteria, e.g. 16 μg/ml for *Pseudomonas aeruginosa*. The intracystic MEPM concentration might not reach the effective level for such bacteria, which may explain why MEPM is not always effective for cyst infection. If cyst infection does not respond to antibiotic therapy with MEPM, we should consider that the intracystic MEPM concentration may be too low for it to be effective against the causative bacteria and should also remember that *Enterococcus* spp. is frequently found in patients with a refractory cyst infection [21].

Fungi were only detected in two episodes in our present series, but we should additionally consider this possibility in patients with refractory infection. There are a few reports of cyst infection induced by fungi from other facilities as well [37–39].

### Our antibiotics strategy for cyst infection at Toranomon Hospital

It is important to identify causative microorganisms in order to avoid empirical therapy, so blood cultures should be performed before administering antibiotics, along with urine cultures in patients with pyuria and cyst fluid cultures after cyst drainage. Antibiotic therapy should be started as soon as possible if cyst infection is suspected because the death rate was reported to be high when infection was caused by multiple microorganisms or when there were multiple infected cysts [21]. If the causative bacteria are identified, appropriate antibiotics should be selected according to the bacteria [21]. If the causative microorganisms are not identified, fluoroquinolones may be used empirically. However, our findings suggest that we should reconsider the empirical use of fluoroquinolones for ADPKD patients with cyst infection [21].

Fluoroquinolones should be used for severe cyst infection, because water-soluble antibiotics, including carbapenem, might not achieve adequate concentrations inside the infected cyst and may therefore not be effective enough. However, water-soluble antibiotics are usually effective for managing mild cyst infection. Indeed, there are some articles that have reported the effectiveness of water-soluble antibiotics for cyst infection in ADPKD [5, 21, 23, 40]. Shankar et al. reported that severe emphysematous polycystic infection by *Klebsiella pneumoniae* was treated by penicillin [40]. We actually administered β-lactamines for 55–73% of the patients with positive cyst content culture or blood culture tests in our hospital [21].

**Fig. 6:**
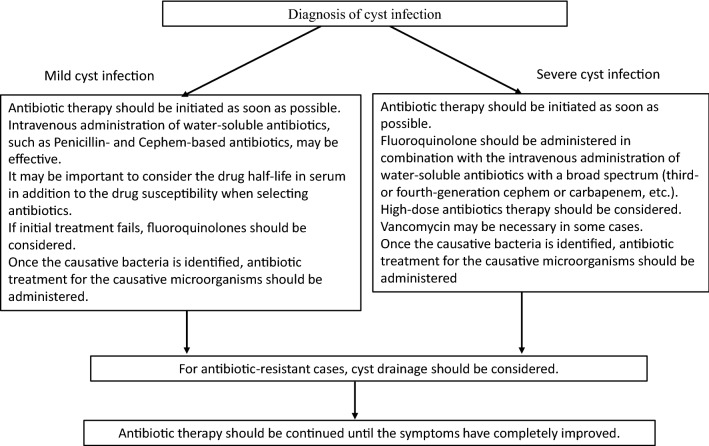
Flowchart of the treatment for cyst infection in ADPKD

Fluoroquinolones should be preferentially administered in cases of severe cyst infection and should be avoided in cases of mild cyst infection as first-line antibiotics in order to prevent their abuse. Below, we suggest antibiotics regimens according to the severity of cyst infection. When determining the severity of cyst infection, we should consider the general condition of the patient, including their blood pressure and body temperature, clinical history, laboratory test findings (e.g. white blood cell count and serum CRP level), and imaging features of infected cysts. Patients with sepsis and disseminated intravascular coagulation (DIC) should be considered to have a severe infection. The definition of mild or severe cyst infection still remains controversial and it is currently up to each individual physician to make such a diagnosis. The clearly defined definition of mild or sever cyst infection should, therefore, be elucidated in the future. We have prepared a flowchart concerning the suggested treatment for cyst infection in ADPKD patients (Fig. 6). This flowchart is based on our experience, and it will be necessary to evaluate its usefulness and generalizability in the future.

### For mild cyst infection

The intravenous administration of water-soluble antibiotics, such as Penicillin- and Cephem-based antibiotics, is usually effective for mild cyst infection. It is important to choose antibiotics with a broad spectrum covering Gram-negative bacteria in terms of drug susceptibility. In addition, the time above the minimum inhibitory concentration (MIC) is an important determinant of the activity of β-lactams. To ensure sufficient time above the MIC of antibiotics in infected cysts, it may be important to consider the drug half-life, as the intracystic antibiotics concentration may increase and be sustained for a longer duration the longer its serum concentration is sustained. It is necessary to accumulate further evidence regarding the association between the drug half-life and its effectiveness against cyst infection. We have described the half-life of some antibiotics in patients with a normal renal function and patient on hemodialysis based on the Medication guidebook for patients on dialysis (Table 5). As shown in Table 5, the half-life of antibiotics differs markedly, especially in patients on dialysis.

**Table 5:** Drug serum half-life of antibiotics

	Half-life in patients with a normal renal function (*h*)	Half-life in patients on hemodialysis (*h*)
Sulbactam/Ampicillin (SBT/ABPC)	SBT 1.0/ ABPC 1.3*	SBT 13.4/ABPC 17.4^a^
Tazobactam/Piperacillin (TAZ/PIPC)	TAZ 0.9/ PIPC 1.0*	TAZ 7.4/PIPC 2.1*
Cefazolin (CEZ)	2.5*	26.4^b^
Cefotiam (CTM)	1.0^c^	2.7^c^
Cefmetazole (CMZ)	1.0 –1.2*	6.2–7.4*
Ceftazidime (CAZ)	1.7^d^	13^c^
Cefotaxime (CTX)	1.0*	2.4*
Ceftriaxone (CTRX)	7–9^c^	12– 24^c^
Cefepime (CFPM)	2 ^d^	18^d^
Flomoxef (FMOX)	0.76*	α; 0.4, β; 17.4*
Meropenem (MEPM)	1.1^c, e^	6–8^c, e^
Imipenem (IPM)	1.0^e^	4.0^c, e^

*Sited from medical package insert of each drug

^a^Blum RA, Kohli RK, Harrison NJ, Schentag JJ. Pharmacokinetics of ampicillin (2.0 grams) and sulbactam (1.0 gram) coadministered to subjects with normal and abnormal renal function and with end-stage renal disease on hemodialysis. Antimicrob Agents Chemotherapy. 1989;33:1470-6

^b^ Fogel MA, Nussbaum PB, Feintzeig ID, Hunt WA, Gavin JP, Kim RC. Cefazolin in chronic hemodialysis patients: a safe, effective alternative to vancomycin. Am J Kidney Dis. 1998;32:401-9

^c^ Bennett WM. Clin Pharmacokinet. Drug Data Handbook 3rd edition. . Auckland: Adis International company; 1998

^d^United States Pharmacopeial Convention : Drug Information for the Health Care Professional 27th edition Vol 1. . 2007

^e^ Aronoff GR. Drug Prescribing in Renal Failure : Dosing Guidelines for Adults and Children 5th edition. Philadelphia: American College of Physicians; 2007

The efficacy of the oral administration of water-soluble antibiotics is still unclear, as the bioavailability of these antibiotics may be low [41, 42]. At present, avoiding the oral administration of water-soluble antibiotics may be best. More cases describing the oral administration of water-soluble antibiotics should be accumulated in the future.

### For severe cyst infection

Lipid-soluble antibiotics should be used for severe cyst infection. However, fluoroquinolone-resistant microorganisms show a high prevalence in cyst infection. Therefore, we suggest that the intravenous administration of water-soluble antibiotics with a broad spectrum be used as well, in combination. Third- or fourth-generation cephem and carbapenem can be promising options for such cases. High-dose antibiotic administration may also be necessary in moribund patients to obtain a higher serum concentration of antibiotics, as high-dose antibiotics are administered to patients with bacterial meningitis [43]. However, high-dose antibiotics therapy may be associated with more frequent side effects than lower doses. We, therefore, need to balance the effectiveness and side effects of high-dose antibiotics therapy and build a consensus concerning the use of high-dose antibiotics for severe cyst infection. Vancomycin may be necessary if combined therapy of lipid-soluble antibiotics and carbapenem is not effective, as *Enterococcus* spp. is a relatively frequently identified causative bacterium.

**Fig. 7:**
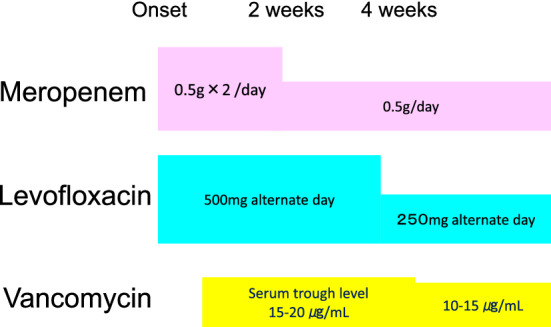
An example of the antibiotics regimen for severe cyst infection in patients on hemodialysis

We have suggested an example antibiotic regimen for severe cyst infection in patients on hemodialysis whose causative bacteria is unknown (Fig. 7). High-dose MEPM and levofloxacin (LVFX) are administered together. If this is not sufficiently effective, vancomycin (VCM) should be added, as *Enterococcus* spp. is a frequent causative bacteria of cyst infection. The dose of antibiotics should be high at first and can later be reduced to a safer dose after the patient’s serious condition is improved.

We evaluated the efficacy of this antibiotic treatment regimen in our hospital. We analyzed the clinical outcome of patients who developed septic shock between 2016 and 2019. During this period, 8 patients died among 15 patients (53.3%) who did not receive combined antibiotic therapy based on our regimen (high dose carbapenem + fluoroquinolones ± Vancomycin). In contrast, only 1 patient died from fungus infection among 8 patients (12.5%) who had received combined antibiotic therapy based on our regimen during the same period. There findings suggest that our strategy may be useful in most patients with severe cyst infection, but we need to accumulate more cases to confirm this. We should also build a consensus concerning the target dose and duration of such intensive antibiotic treatment. If the causative bacteria are able to be identified, antibiotic therapy should be adjusted according to the bacteria. However, we should be careful about superinfection due to the presence of additional microorganisms, since superinfections may occur in patients with a severe cyst infection [21].

### Cyst drainage

The indication of cyst drainage is still controversial [44]. No evidence supporting the indication of cyst drainage has been gathered. Generally speaking, cyst drainage is recommended if a fever persists for one to two weeks despite appropriate antimicrobial therapy [45]. Cyst drainage may also be recommended for large, infected cysts exceeding 5 cm in diameter [5]. We have suggested adaptation criteria for cyst drainage in ADPKD cases with cyst infection (Table 6). We consider severe cyst infection should be indicated for cyst drainage because the amount of bacteria is large and it can be more serious and even fatal when it relapses. Patients demonstrating cyst infection with many repeat episodes are also recommended to undergo cyst drainage. We performed 1,038 cyst drainage procedures from 2004 to 2019. We experienced several cases with severe adverse events related to cyst drainage (Table 7). Given that cyst drainage can be associated with severe adverse events, we should establish adaptation criteria for cyst drainage in order to justify this procedure. The definitive operative procedure has not yet been established either. We usually place an indwelling catheter in the infected cysts for one week, and wash out the cyst contents with saline solution every day. The timing for cyst drainage also remains controversial. We believe that we should avoid performing cyst drainage when patients are in a poor condition because the patients’ condition can worsen and become fatal just after cyst drainage due to the effects of surgical invasion. Therefore, we usually perform cyst drainage after patients first show an improvement in their overall condition even if antibiotic therapy is slightly effective. There are also some patients who do not respond to antibiotic therapy at all and, therefore, need to receive cyst drainage as soon as possible. We should continue to explore the best surgical procedures and optimal timing for performing cyst drainage in the future.

**Table 6:** Suggested criteria for cyst drainage in cyst infection in ADPKD

Infection resistant to antibiotics therapy (a fever that persists for 1–2 weeks despite appropriate antimicrobial therapy)
Large infected cyst exceeding 5 cm in diameter
Severe cyst infection (sepsis, disseminated intravascular coagulation [DIC], etc.)
Cyst infection with many repeat episodes

**Table 7:** Severe adverse events related to cyst drainage in Toranomon Hospital (January 2014 to June 2019)

	Number of events	Percentage of all cyst drainage (%)
Plueroperitoneal communication	7	0.66
Bile fistula	6	0.57
Peritonitis	5	0.47
Pneumothorax	4	0.38
Severe intracystic bleeding	2	0.19
Bile duct hemorrhagic cholangitis	1	0.09
Gastrointestinal perforation	1	0.09
Sudden death	3	0.28
All	29	2.75

Number of patients who underwent cyst drainage: 1054 between January 2014 to June 2019

### Surgical resection

Nephrectomy or partial hepatectomy is performed for refractory cyst infection in which cyst drainage is technically impossible. However, nephrectomy or partial hepatectomy can be associated with severe adverse events, so surgical indications should be considered carefully.

### Effectiveness of renal transarterial embolization for renal cyst infection

We previously reported that the incidence of renal cyst infection might decrease if adequate renal volume (RV) reduction can be achieved by renal TAE [10, 23]. From 2004 to 2016, 807 patients underwent renal TAE in our hospital, 16 of whom (2.0%) had definite renal cyst infection. A total of 61 patients had definite renal cyst infection in our hospital from 2004 to 2016. Among them, 16 had undergone renal TAE before, while 45 had not undergone renal TAE before. In contrast, 379 patients underwent hepatic TAE in our hospital from 2004 to 2016, 48 of whom (12.7%) had a definite hepatic cyst infection. The ratio of patients who had a definite renal cyst infection among those who received renal TAE was significantly lower than the ratio of patients who had a definite hepatic cyst infection among those who received hepatic TAE (2.0% vs. 12.7%, *p*< 0.0001). This fact might support our hypothesis that renal TAE prevents renal cyst infection in ADPKD. As mentioned above, renal cysts are infected through the hematogenous spread of bacteria. Renal TAE occludes renal arteries as much as possible. If the renal blood flow is shut off completely after renal TAE, renal cyst infection can theoretically be prevented. However, a few patients still suffer from renal cyst infection. Indeed, in our series, 16 patients had definite renal cyst infection from 2004 to 2016. This may be because the renal blood flow was not shut completely after renal TAE. Indeed, some patients still release small amounts of urine even after renal TAE. In addition, the nutrition status improves after renal TAE as the food intake increases [46], which may also help prevent cyst infection.

However, we experienced patients with severe renal cyst infection that occurred just after renal TAE in whom renal cyst infection might have still been active [47]. The renal blood flow decreases after renal TAE, so antibiotics may be unlikely to reach the infected renal cysts through the blood flow, and the antibiotics concentration in infected cysts may, therefore, not achieve the level needed in such patients after renal TAE. The cyst infection may consequently worsen after renal TAE. Renal cyst infection can progress rapidly after renal TAE, and it should not be performed in patients with active cyst infection. Further studies are needed in order to clarify whether or not renal TAE can prevent renal cyst infection in ADPKD patients.

In contrast to renal TAE, hepatic TAE may not prevent hepatic infection at all. In addition, hepatic TAE was not significantly associated with death from cyst infection, but it was significantly associated with a longer hospitalization duration in our series [23]. This may be because hepatic arteries are occluded only partially by hepatic TAE, and the invasion of bacteria into hepatic cysts through hepatic arteries is thus not prevented. We were unable to assess the impact of hepatic TAE on the incidence of cyst infection in that study, so further investigations will be needed to determine whether or not hepatic TAE influences the risk of cyst infection.

### Prevention of cyst infection

As mentioned above, hematogenous spread via bacterial translocation in the intestine is considered to be the main cause of cyst infection [21]. Most causative bacteria are enterobacteria, so the intestinal microbiome may be important for cyst infection. In fact, intestinal probiotic bacteria, such as lactobacillus and bifidobacteria, are not causative bacteria of cyst infection [21]. The intestinal microbiome and innate immune system cooperate in the eradication of bacterial infection [48, 49]. Probiotic administration may be effective for preventing cyst infection, as probiotics administration has been shown to be effective for preventing colitis [50]. Furthermore, specific probiotic lactobacillus strains reportedly influence the host immune system by promoting immunoglobulin A (IgA) production [51]. The role of intestinal microbiome on cyst infection in ADPKD is unknown, and further research on this topic is needed.

Some patients developed cyst infection after they caught a cold or enteritis. Bacterial translocation may be more likely to occur in patients with a poor physical condition. In fact, we experienced a patient who developed a cyst infection after enteritis, and his causative bacteria of the cyst infection was exactly the same as that of enteritis [26]. This underscores the importance of maintaining a good physical condition. The nutrition status may also be important, as it may be related to immunity [52, 53]. It is known that malnutrition in patients on hemodialysis is associated with impaired immunity [54]. Patients with repetitive cyst infection are often malnourished with low albuminemia, so it may be important to maintain a good nutrition state in order to ameliorate impaired immunity and prevent cyst infection. More research on this topic is needed as well.

Renal TAE may be effective for preventing cyst infection in terms of blocking the hematogenous spread of bacteria and improving the nutrition status, and we should continue our research on this. Massive hepatomegaly is the strongest factor influencing severe cyst infection, so it is important to prevent massive enlargement of the liver.

## Limitation

Our treatment strategy is based on our experience at Toranomon Hospital. The clinical characteristics of the patients with cyst infection, causative bacteria for cyst infection and their susceptibility to antibiotics vary among facilities. Therefore, our strategies cannot be generalized. The treatment strategy should be optimized to each facility according to the background characteristics.
